# Transplantation of Saccharomyces cerevisiae Rmd9p peptide into mammalian mitochondrial IF2 substitutes for the IF1 function in Escherichia coli

**DOI:** 10.1099/mic.0.001689

**Published:** 2026-03-27

**Authors:** Jitendra Singh, Amit Kumar Sahu, Umesh Varshney

**Affiliations:** 1 Department of Microbiology and Cell Biology, Indian Institute of Science, Bangalore 560012, India

**Keywords:** IF1, mitochondrial IF2, Rmd9p, translation initiation factors

## Abstract

Mitochondrial translation machinery exhibits similarities with the bacterial translation apparatus. Of the three bacterial translation initiation factors (IF1, IF2 and IF3), two (IF2 and IF3) have homologues in mitochondria (mtIF2 and mtIF3). A high conservation of decoding nucleotides in the ribosomal A-site suggests relevance of IF1-like proteins in mitochondria. The mitochondrial translation machineries have evolved with different solutions for the IF1 function. However, in *Saccharomyces cerevisiae*, the identity of such a protein remains unknown. Here, based on sequence alignment with human mtIF2, we deduced that Rmd9p may contribute to an IF1-like function in *S. cerevisiae*. Our genetic analyses show that Rmd9p is required for mitochondrial translation. In addition, we show that a sequence from Rmd9p, pivotal for its mitochondrial function, when inserted into mtIF2, substitutes for both the IF2 and IF1 functions in an established model of *Escherichia coli*. Interestingly, while the mutations at the critical residues in the Rmd9p peptide compromise the IF1 function, the mutant peptide is still able to support *E. coli* growth, suggesting that the structure (rather than the precise sequence) of the IF1-like insert domain in mitochondrial IF2 plays a major role in the recognition of the decoding nucleotides in the ribosomal A-site.

## Introduction

Mitochondria are of ubiquitous occurrence in eukaryotic cells and possess an independent gene expression machinery. Notably, the mitochondrial translational apparatus is more closely related to the bacterial system than to the cytosolic one. Although mitochondrial ribosomes have diverged substantially across eukaryotic lineages, core functional components like the decoding centre and peptidyl-transferase centre remain highly conserved [1]. Unlike bacteria which possess three translation initiation factors (IF1, IF2, and IF3), mitochondria are known to possess homologues of only two canonical bacterial translation initiation factors, IF2 and IF3. A dedicated homologue of IF1, an initiation factor conserved across all three domains of life, is conspicuously absent from the known mitochondrial translation machinery. In bacteria, IF1 is a small OB-fold protein of the S1 family that binds 30S ribosomal subunit A-site, contacts highly conserved decoding-centre nucleotides (G530, A1492 and A1493) and occludes aminoacyl-tRNA entry into the A-site during initiation to promote accurate and productive initiation complex formation [2]. While an independent IF1 protein is absent in the mitochondrial system, different organisms assign IF1 functions to distinct proteins. Using *Escherichia coli* as a successful model, we showed that in vertebrate mtIF2, a 37 amino acid insert assumes the role of IF1 [3]. Likewise, in *Trypanosoma brucei*, a C-terminal extension (CTE) in mtIF3 may perform the function of mtIF1 [4]. Both the mtIF2 insert and mtIF3-CTE exhibit distinct structural folds compared to IF1. Nonetheless, they interact with the conserved decoding nucleotides, indicating the importance of IF1 function in translation initiation [4,5].

*Saccharomyces cerevisiae* is an important model to study mitochondrial translation. In this yeast, mitochondria harbour 74S ribosomes (mitoribosomes) responsible for synthesizing 8 proteins. The genes *ifm1* and *aim23* encode IF2 and IF3 homologues, respectively, which are crucial for optimal mitochondrial translation [6,8]. The conservation of the decoding centre in the 74S mitoribosome provisions for the presence of an IF1-like protein in *S. cerevisiae*. However, unlike its vertebrate counterpart, yeast mtIF2 lacks an insert domain, and yeast mtIF3 does not possess an unusually long CTE found in *T. brucei* [9].

Earlier studies have suggested Aep3p, Rsm28p, Msc6p and Rmd9p as potential functional homologues or candidates for mitochondrial IF1 in *S. cerevisiae* [9,10]. Among these, Rsm28p (mS46) is a mitoribosomal protein located on the mitoribosomal smaller subunit (mtSSU) head, while the remaining candidates – Aep3p, Msc6p and Rmd9p – belong to the pentatricopeptide repeat (PPR) family. PPR proteins are known for their involvement in several aspects of mitochondrial gene expression [11,13]. Notably, Aep3p and Msc6p are shown to interact with mtIF2; and Aep3p and Rmd9p interact with mtSSU [10,14,17]. While Rsm28p is dispensable for overall mitochondrial translation, it is strictly required for efficient translation of Cox1, Cox2 and Cox3 mRNAs [18]. Mutations in Ifm1p, Rmd9p or Fmt1p (deletion) in combination with Rsm28p show the synthetic respiratory defect [17]. Msc6p localizes in the mitochondrial matrix, and its overexpression partially suppresses respiratory lethality caused by qrs1/her2 glutamyl-tRNA amidotransferase mutants [19,20] While Msc6p does not co-sediment with the mitoribosome, it interacts with mtIF2, indicating its role in mitochondrial translation [10]. Aep3p is an inner mitochondrial membrane protein, which stabilizes ATP6/8 mRNA [21] and is required for translation of ATP8p [22]. It also interacts with mtIF2 and enhances its ability to bind unformylated i-tRNA under methionyl-tRNA formyltransferase (FMT1) deficiency [15]. Rmd9p stabilizes mitochondrial mRNAs by binding to their 3′-untranslated regions and plays a role in the biogenesis of the small ribosomal subunit by contributing to the 15S rRNA 3′ processing [16,23, 24].

In this study, we have primarily focused on elucidating the potential role of Rmd9p as a candidate for the IF1. We show that a sequence in Rmd9p substitutes for the role of an IF1-like insert in mtIF2.

## Methods

### Strains and growth conditions

Yeast and *E. coli* strains used in this study are listed in Table 1. For yeast, cultures were grown in yeast extract-peptone (YP) broth or YP agar plates containing 2% yeast extract (Gibco), 1% peptone (Gibco) and 2% glucose or 3% glycerol as a carbon source. Cultures were supplemented with geneticin (G418, 200 µg ml^−1^) as required. For selective media, 0.67% yeast nitrogenous base without amino acids (Sigma) was mixed with 0.192% yeast dropout mix without uracil or histidine (Sigma) and 2% glucose or 3% glycerol as a carbon source. For *E. coli*, cultures were grown in Luria–Bertani (LB) broth or LB agar plates containing 1.8% Bacto agar (Gibco). Cultures were supplemented with chloramphenicol (Cm, 10 or 30 µg ml^−1^), or tetracycline (Tet, 7.5 µg ml^−1^) as required.

**Table 1.** List of *S. cerevisiae* and *E. coli* strains used in this study

S.N.	Strain	Description	Reference
**Yeast strains**			
1	CW252	W303 derivative with genotype *MATα, ade2; trp1; ura3; leu2; his3*, ρ^+^, intronless	[41]
2	CW252 *Δrmd9::kanMX*	CW252 with *rmd9* gene disrupted with *kanMX* cassette	[24]
***E. coli* strains**			
3	TG1	*E. coli* K12 derivative (*supE hsd*Δ5 *thi* Δ(*lac-proAB*) F` [*traD*36 *proAB*^+^*lacI*^q^*lacZ*ΔM15])	[42]
4	MG1655*ΔinfB*::*kan*/pACDH IF2	*E. coli* MG1655 deleted for IF2 gene with *kan*^R^ cassette under wild-type IF2 support	[3]
5	TG1*ΔinfA*::*cm/*pTrc IF1	*E. coli* TG1 deleted for IF1 gene with *cm*^R^ cassette under wild-type IF1 plasmid support	[3]
6	TG1*ΔinfB*::*kan/p*ACDH mtIF2	*E. coli* TG1 deleted for IF2 gene with *kan*^R^ cassette under bovine mtIF2 plasmid support	[3]
7	TG1*ΔinfB*::*kan/p*ACDH cRmd9^WT^	*E. coli* TG1 deleted for IF2 gene with *kan*^R^ cassette under Rmd9 chimaera (wild-type) plasmid support	This study
8	TG1*ΔinfB*::*kan/p*ACDH cRmd9^MUT^	*E. coli* TG1 deleted for IF2 gene with *kan*^R^ cassette under Rmd9 chimaera (mutant) support	This study
9	TG1*ΔinfB*::*kan/p*ACDH cRsm28	*E. coli* TG1 deleted for IF2 gene with *kan*^R^ cassette under Rsm28 chimaera plasmid support	This study
10	TG1*ΔinfB*::*kan/p*ACDH cMsc6	*E. coli* TG1 deleted for IF2 (with kan^R^) and IF1 (with cm^R^) genes with Msc6 chimaera support	This study
11	TG1*ΔinfB*::*kan ΔinfA*::*cm/p*ACDHmtIF2	*E. coli* TG1 deleted for IF2 and IF1 genes with kan^R^ and cm^R^ cassettes with bovine mtIF2 support	[3]
12	TG1*ΔinfB*::*kan ΔinfA*::*cm/p*ACDH cRmd9^WT^	*E. coli* TG1 deleted for IF2 gene with *kan*^R^ cassette and IF1 gene with cm^R^ cassette under Rmd9 chimaera (wild-type) plasmid support	This study
13	TG1*ΔinfB*::*kan ΔinfA*::*cm/p*ACDH cRmd9^MUT^	*E. coli* TG1 deleted for IF2 gene with *kan*^R^ cassette and IF1 gene with *cm*^R^ cassette under Rmd9 chimaera (mutant) plasmid support	This study
14	TG1*ΔinfB*::*kan ΔinfA*::*cm/p*ACDH cRsm28	*E. coli* TG1 deleted for IF2 gene with *kan*^R^ cassette and IF1 gene with *cm*^R^ cassette under Rsm28 chimaera plasmid support	This study
15	TG1*ΔinfB*::*kan ΔinfA*::*cm/p*ACDH cMsc6	*E. coli* TG1 deleted for IF2 gene with *kan*^R^ cassette and IF1 gene with *cm*^R^ cassette under Msc6 chimaera plasmid support	This study

## Plasmids, cloning and mutagenesis of Rmd9

Various plasmids used/generated in the study are listed in Table 2. Rmd9 ORF, along with its native promoter, was PCR-amplified from *S. cerevisiae* CW252 genomic DNA and cloned into pRS416 TEF vector after SacI and XhoI digestion. For the generation of the Rmd9 (W457A/R460A/F465A) mutant, first, W457A and R460A mutations were introduced together (W457A/R460A mutant was also used), sequence verified, and then, F465A mutation was introduced using a standard site-directed mutagenesis protocol [25]. Both wild-type and the mutant genes were also cloned under the TEF promoter in pRS416 TEF [26] or pRS426 TEF [26] vectors at XbaI and XhoI sites. Single Rmd9 mutants (W457A, R460A and F465A) were also generated.

**Table 2.** List of plasmids used in this study

S.N.	Plasmid	Description	Reference
1	pRS416 TEF	YX-type (expression) with low copy number shuttle vector	[26]
2	pRS426 TEF	YX-type (expression) with low copy number shuttle vector	[26]
3	pRS416 Rmd9	Rmd9 ORF with intrinsic promoter cloned into pRS416 TEF at SacI and XhoI sites	[24]
4	pRS416 TEF Rmd9	Rmd9 ORF cloned under TEF promoter into pRS416 TEF at XbaI and XhoI sites	This study
5	pRS416 Rmd9 (W457A)	Rmd9 ORF (W457A) with intrinsic promoter cloned into pRS416 TEF at SacI/XhoI sites	This study
6	pRS416 Rmd9 (R460A)	Rmd9 (R460A) ORF with intrinsic promoter cloned into pRS416 TEF at SacI/XhoI sites	This study
7	pRS416 Rmd9 (F465A)	Rmd9 (F465A) ORF with intrinsic promoter cloned into pRS416 TEF at SacI/XhoI sites	This study
8	pRS416 Rmd9 (W457A/R460A)	Rmd9 (W457A/R460A) ORF with intrinsic promoter cloned into pRS416 TEF at SacI/XhoI sites	This study
9	pRS416 Rmd9 (W457A/R460A/F465A)	Rmd9 (W457A/R460A/F465A) ORF with intrinsic promoter cloned into pRS416 TEF at SacI/XhoI sites	This study
10	pRS416 Rmd9 TEF (W457A/R460A/F465A)	Rmd9 (W457A/R460A/F465A) ORF cloned under TEF promoter into pRS416 TEF at XbaI/XhoI sites	This study
11	pRS426 Rmd9 TEF (W457A/R460A/F465A)	Rmd9 (W457A/R460A/F465A) ORF cloned under TEF promoter into pRS426 TEF at XbaI/XhoI sites	This study
12	pACDH mtIF2	Bovine mtIF2 cloned under NdeI and HindIII sites in pACDH vector	[3]
13	pACDH cRmd9^WT^	pACDHmtIF2 derivative, where mtIF2 insert is replaced with sequence from Rmd9	This study
14	pACDH cRmd9^MUT^	pACDHmtIF2 derivative, where mtIF2 insert is replaced with sequence from Rmd9 (W457A/R460A/F465A)	This study
15	pACDH cRsm28	pACDHmtIF2 derivative, where mtIF2 insert is replaced with sequence from Rsm28	This study
16	pACDH cMsc6	pACDHmtIF2 derivative, where mtIF2 insert is replaced with sequence from Msc6	This study

## Transformation of yeast cells

The transformations were performed by the lithium acetate method [27]. Briefly, mid-log culture was pelleted and washed with sterile Milli-Q once. Then, the pellet was resuspended in 0.1 M lithium acetate and divided into small aliquots depending on the initial culture volume. After centrifugation, the following components were added in an orderly manner: 240 µl of polyethylene glycol (50%, PEG-3350), 36 µl of 1M lithium acetate, 10 µl salmon sperm DNA (10 mg ml^−1^), 72 µl Milli-Q and 2 µl plasmid (~100 ng) or 2 µl linear DNA (~500 ng). The components were mixed by vortexing and kept at 30 °C for 30 min with constant shaking followed by a heat shock at 42 °C for 45 min. The cell pellet was either resuspended in Yeast extract-Peptone-Dextrose (YPD) media and kept for recovery for 1 h at 30 °C before plating on a G418 plate or resuspended in sterile Milli-Q and directly plated on selective plates.

### Generation of chimaeras

All chimaeras were generated by inverse PCR using pACDH mtIF2 as a template [3] and are listed in Table 2. A primer set was designed to replace the mtIF2 insert region with the respective sequence present in the chimaeras. PCR was performed using Q5 DNA polymerase (NEB) with annealing at 69 °C for 25 cycles. The PCR product was digested with DpnI (Fermentas), and the replacement was confirmed by Sanger’s sequencing method.

### Transduction in *E. coli*

Transductions were conducted to generate IF1, IF2 knockouts under bovine mtIF2 or *rmd9* chimaera support in *E. coli*. IF2 knockouts were performed using P1 donor lysate raised on MG1655*ΔinfB::kan*/pACDH IF2 strain, and IF1 knockouts were performed using P1 donor lysate raised on TG1*ΔinfA::cm*/pTrcIF1 strain as follows. The overnight culture of *E. coli* recipient strain (2 ml) was mixed with 10 µl of 1 M CaCl_2_ and 100 µl of P1 phage raised on the donor strain. The mixture was incubated at 37 °C for 20 min. Then, 100 µl of 0.5 M sodium citrate was added and vortexed to stop the phage infection. The cell pellet was resuspended in 5 ml LB broth and 100 µl of 0.5 M sodium citrate and kept in recovery for 1 h. After recovery, the cells were plated on LB agar containing appropriate antibiotics (25 µg ml^−1^ for kanamycin and 10 µg ml^−1^ chloramphenicol). The knockouts were confirmed by PCR.

### Rmd9p purification and antibody generation

Rmd9 ORF was PCR amplified using NdeI and XhoI site containing primers and cloned into pET28b vector at NdeI and XhoI sites to generate an N-terminally His_6_-tagged clone. His-tagged protein was partially purified from the BL21 strain using one round of Ni-NTA chromatography. A total of 3 mg protein was resolved on SDS-PAGE and the band corresponding to Rmd9p was excised. A very thin paste of the excised band with Freund’s incomplete adjuvant was prepared by crushing it with a mortar and pestle. The paste was injected into a female rabbit, and the first booster was given after 28 days. A second booster was given 14 days post-first booster. Finally, 14 days post-second booster, blood was collected (∼25 ml) and centrifuged at 5,000 r.p.m. for 20 min. Clear serum was transferred to fresh microfuge tubes and stored at −20 °C.

### Total protein isolation and immunoblotting

The mid-log phase yeast culture was harvested and washed with Milli-Q water. The cell pellet equivalent to 0.6 OD was resuspended in 75 µl of solubilization buffer (1.8 N NaOH, 1 M *β*-mercaptoethanol and 10 mM PMSF), vortexed and diluted with 500 µl Milli-Q water. Proteins were precipitated with 575 µl of 50% TCA. The precipitate was first washed with 1.5 ml of 0.5 M Tris base and 1 ml of Milli-Q water, respectively. The pellet was resuspended in 25 µl of gel sample buffer [2% SDS, 10% glycerol, 60 mM Tris (pH 6.8), 2.5% *β*-mercaptoethanol and 0.02% bromophenol blue], heated at 95 °C for 5 min and loaded on 12% polyacrylamide gel. After the run, proteins were transferred on PVDF membrane (G Biosciences) followed by blocking in 5% skim milk at room temperature for 2 h. The blot was incubated with primary antibody (1:3,000, anti-rmd9p polyclonal, in house; 1:10,000 anti-IF2 polyclonal, in house) overnight at 4 °C and washed thrice with 1× Tris Buffered Saline Tween-20 (TBST, 20 mM Tris–HCl (pH 7.5), 0.9% NaCl and 0.2% v/v Tween-20) and then incubated with the secondary antibody (1:5,000; anti-rabbit IgG-HRP, Genei) in 1× TBST for 2 h. The blot was washed again thrice with 1× TBST, developed using ECL reagent (Millipore) and scanned in ChemiDoc (GE).

### RNA isolation and Northern blotting

For isolation of total RNA, a saturated yeast culture was sub-cultured in 5 ml broth and grown till OD_600_ of ~1–2. Cells were pelleted and washed once with DEPC-treated water. Pellet was resuspended in 500 µl of Tri reagent (Sigma), and ~200 µl of acid-washed beads was added to facilitate cell lysis and disruption of the cell membrane. Bead beating was performed for four cycles, each lasting 45 s with in-between incubation in ice for 5 min after each cycle. Subsequently, 100 µl of chloroform was added, and the mix was incubated at room temperature for 1 min prior to precipitation with alcohol. For Northern blot, 6 µg of total RNA was subjected to electrophoresis on a 2% agarose gel using 1× Tris-Borate-EDTA buffer (TBE, 89 mM Tris, 89 mM boric acid and 2 mM Na_2_EDTA) for 14 h at 70 V in cold. Following gel electrophoresis, the RNA was transferred from the agarose gel to a nylon membrane (G-Biosciences) at a constant voltage of 5 V for a duration of 3:30 h using a semi-dry trans-blot apparatus (Bio-Rad). The RNA was crosslinked by UV treatment and kept at blocking in 1× prehybridization buffer [2× prehybridization buffer contains 10× Denhardt reagent [0.2% Ficoll 400 (w/v), 0.2% polyvinylpyrrolidone (w/v) and 0.2% BSA], 10× sodium chloride sodium citrate (SSC, 1.5 M NaCl, 0.3 M sodium citrate, 30 mg/100 ml yeast RNA, 1% SDS) at 65 °C. The 5′ end labelled probe was added to the membrane and allowed to hybridize with the immobilized RNA overnight at 43 °C. Following hybridization, the membrane was subjected to a series of three wash steps to remove any unbound or non-specifically bound probe. The wash buffers used were as follows: buffer I (4× SSC and 0.1% SDS), buffer II (2× SSC and 0.1% SDS) and buffer III (1× SSC). Each wash step was carried out for a duration of 30 min at 43 °C. Finally, the membrane was exposed to a phosphor-imager screen and analysed using a BioImage Analyzer (FLA5100, Fuji Film).

### Labelling of mitochondrial translation products

The protocol was adapted from [28]. Briefly, yeast strains were grown till OD_600_ of 1–2 in appropriate growth media containing 2% galactose as a carbon source. Cells equivalent to 0.6 OD_600_ were pelleted and washed with 500 µl of reaction buffer [40 mM potassium phosphate buffer (pH 6.0), 2% galactose]. The pellet was resuspended again in the reaction buffer, and 10 µl aqueous solution of freshly prepared cycloheximide (10 mg ml^−1^) was added to inhibit cytosolic translation. After incubation for 2:30 min at room temperature, 4.5 µl of S^35^-methionine (10 mCi ml^−1^) was added and incubated for an additional 15 min. The cells were pelleted and 75 µl of solubilization buffer (1.8 N NaOH, 1 M *β*-mercaptoethanol and 10 mM PMSF) was added to stop the reaction. The reaction was vortexed and diluted with 500 µl Milli-Q water. Total proteins were precipitated with the addition of 575 µl of 50% TCA and incubated in ice for 10 min. The pellet was first washed with 1.5 ml of 0.5 M Tris base followed by 1 ml of Milli-Q water and resuspended in 25 µl of gel sample buffer [2% SDS, 10% glycerol, 60 mM Tris (pH 6.8), 2.5% *β*-mercaptoethanol and 0.02% bromophenol blue] and loaded on 17.5% polyacrylamide gel. The gel was directly exposed to a phosphor-imager screen and analysed on BioImage Analyzer (FLA5100, Fuji Film).

### Blue-native PAGE

Blue-native PAGE (BN-PAGE) protocol was adapted from [29]. The yeast strains were subcultured and grown till OD_600_ of 1–2 in YPD media. The cells were pelleted and washed once with Milli-Q followed by buffer A [2 ml g^−1^ cell wet weight; 100 mM Tris (pH 8.8), 10 mM DTT] and incubated at 30°C for 10 min. The pellet was washed with 1.2 M sorbitol and resuspended in cell wall digestion buffer [6.7 ml g^−1^ cell wet weight; 20 mM potassium phosphate buffer (pH 7.4), 0.6 M sorbitol and zomolyase-50T (4 mg/gm cells)] and kept at 30 °C for 1 h with constant shaking. Spheroplasts were harvested by centrifugation and resuspended in homogenization buffer [1 ml/0.15 gm cell wet weight; 0.6 M sorbitol, 10 mM Tris (pH 7.4), 1 mM Na_2_EDTA and 1 mM PMSF]. The suspension was transferred to a pre-cooled Dounce homogenizer, and 15 strokes were applied for cell lysis. After homogenization, cell debris and nuclei were removed by centrifugation at 2,200 ***g*** for 5 min, and the supernatant was again homogenized and centrifuged at 17,000 ***g*** for 15 min; the pellet now containing crude mitochondria was washed once and then resuspended in SH buffer [0.6 M sorbitol, 20 mM HEPES buffer (pH 7.4)]. A 250 µg total protein aliquot of crude mitochondria was pelleted and resuspended in 40 µl of aminocaproic acid buffer [1.5 M aminocaproic acid, 50 mM Bis-Tris (pH 7.0), 1× protease inhibitor cocktail]. For the extraction of OXPHOS complexes, 5 µl of 10% *N*-dodecyl *β*-d-maltoside was added in a 1:2 protein to detergent ratio and incubated in ice for 10 min followed by centrifugation at 35,000 ***g*** for 15 min at 4 °C. To the supernatant, 5 µl of glycerol (50%, v/v) and 2 µl of 10× native loading buffer [750 mM aminocaproic acid, 50 mM Bis-Tris (pH 7.0), 0.5 mM Na_2_EDTA and 5% CBB G250] was added and 40 µl loaded on a 4–16% native gradient gel. After running at 70 V for 1 h at room temperature using anode buffer [10 mM Tris buffer (pH 7.0)] and blue cathode buffer [0.02% CBB G250, 10 mM tricine, 3 mM Bis-Tris (pH 7.0)], the cathode buffer was changed with light cathode buffer [10 mM tricine, 3 mM Bis-Tris (pH 7.0)] and the gel was run at 40 V at 4 °C. The gel was visualized by Coomassie staining and de-staining.

### Structural analyses

Structural analysis was performed using the AlphaFold 3 server, and the structures were analysed using PyMOL.

## Results

### Rmd9p is a potential IF1 candidate in yeast mitochondria

**Fig. 1.**
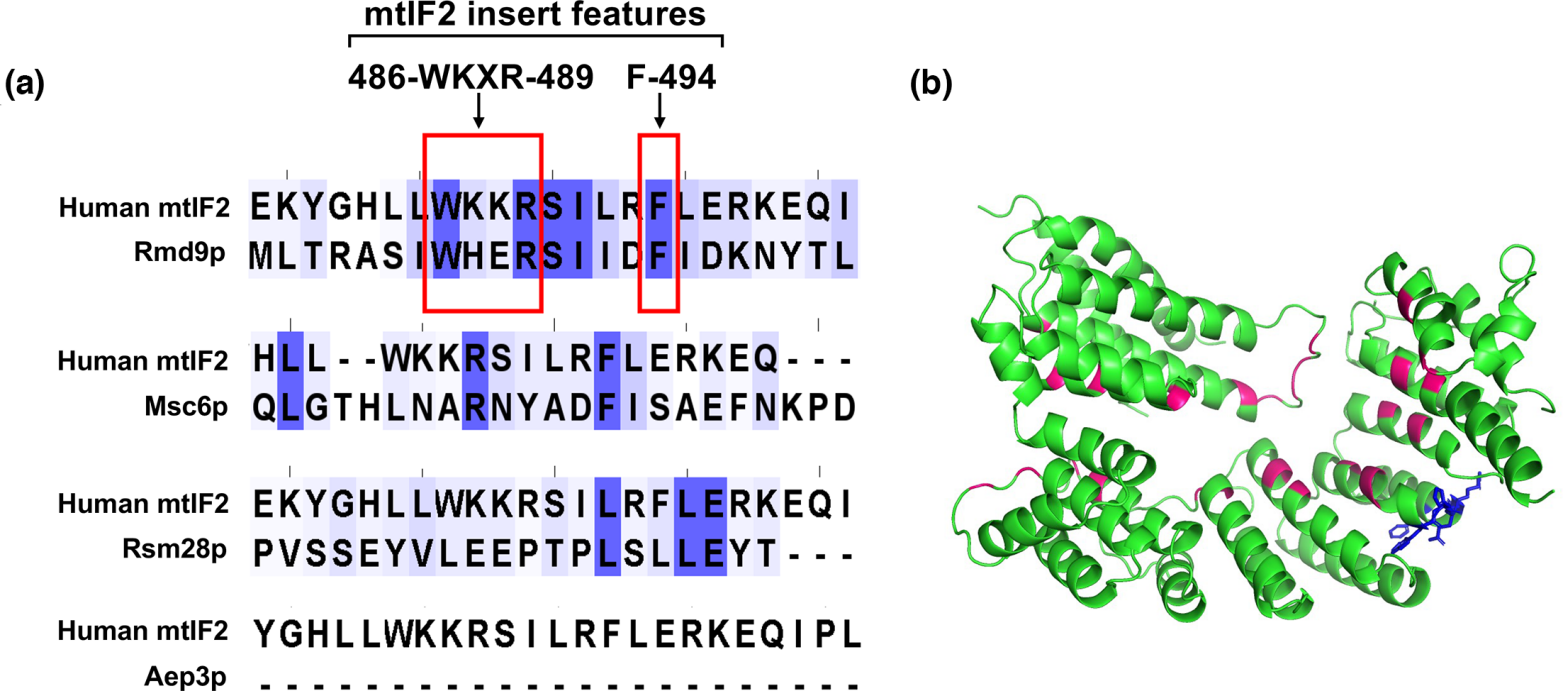
Rmd9p is an IF1 candidate in yeast mitochondria. (**a**) Sequence alignment of human mtIF2 with literature-based IF1 candidates in yeast mitochondria, highlighting features of mtIF2 insert (486-WKXR-489 motif and F-494) on top. (**b**) Structure of Rmd9p highlighting residues interacting with the mRNA 3′-UTR (in pink) and residues homologous to mtIF2 insert features (in blue).

Within mitochondria, the assignment of IF1-like function relies on the interaction between decoding nucleotides and mtIF2 insert domain (in vertebrates) or mtIF3 CTE (in *T. brucei*). While the role of mtIF3 CTE in *T. brucei* is supported primarily by structural data [4], the function of mtIF2 insert in vertebrates is strongly supported by both structural and genetic studies employing *E. coli* as a successful model [3,5]. In human mtIF2, two conserved features have been identified: a Trp-Lys-X-Arg motif (residues 486–489) and an aromatic side chain (residue 494). These features are crucial for interaction with the decoding nucleotides [5]. To identify the protein that plays an IF1-like function in yeast mitochondria, we searched for these features in yeast IF1 candidates by aligning them with human mtIF2 and found a better representation of the features in Rmd9p than in the other three (Aep3p, Rsm28p and Msc6p) candidates (Fig. 1a). Rmd9p contains a Trp-His-Glu-Arg motif (residues 457–460) and an aromatic side chain (F465). Notably, these residues are situated away from the region that interacts with the mRNA 3′-untranslated region (UTR) (Fig. 1b). The presence of these conserved features in Rmd9p makes it an important candidate for an IF1-like function in yeast mitochondria.

### Rmd9p is crucial for mitochondrial translation

**Fig. 2.**
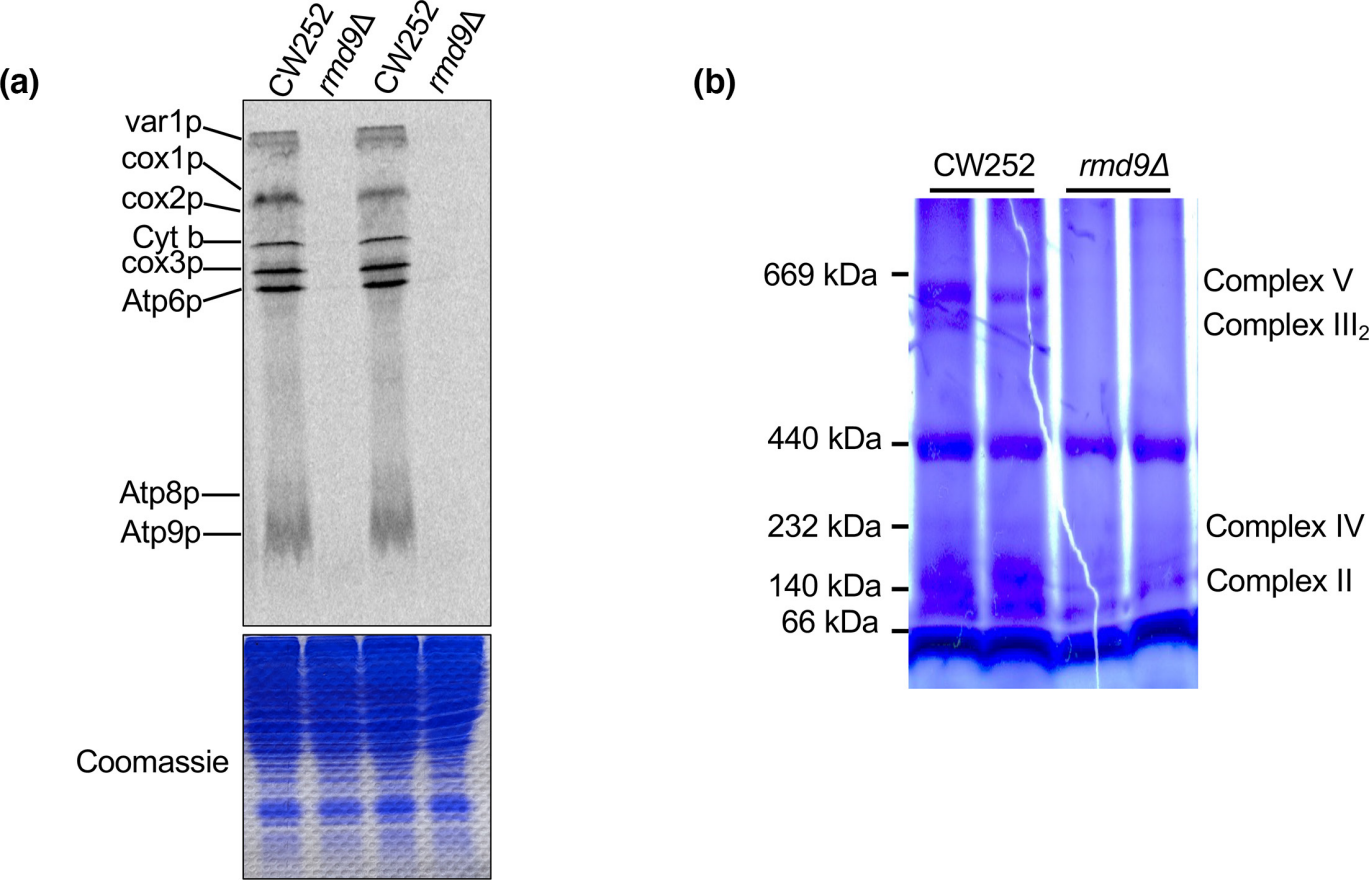
Rmd9p is required for mitochondrial translation. (**a**) S^35^-Methionine labelling of mitochondrial products in CW252 wild-type and *rmd9* deletion strains. (**b**) BN-PAGE analysis to visualize OXPHOS complexes in CW252 wild-type and rmd9 deletion strains.

Rmd9p is required to maintain proper mitochondrial function. When the Rmd9p gene was knocked out, the resulting strain was unable to grow on a non-fermentable carbon source [16]. Previous research has shown that Rmd9p plays a role in mitochondrial mRNA stability by binding to the conserved dodecameric element found on 3′-UTRs [23]. However, the specific role of Rmd9p in mitochondrial translation is not fully understood. To investigate this further, we carried out S^35^-methionine labelling of mitochondrial translation products in both the wild-type (CW252) and the *rmd9* knockout strains. The results revealed a significant impairment in the translation of all mitochondrially encoded proteins in the *rmd9* knockout strain compared to the wild-type strain (Fig. 2a). Considering that most of the mitochondrially encoded proteins, in general, contribute to the OXPHOS system, it was reasonable to assume that any defect in mitochondrial translation would also impact the assembly of OXPHOS complexes. Thus, we investigated the status of OXPHOS complexes using BN-PAGE. As anticipated, the *rmd9* knockout strain showed the absence of complexes containing mitochondrially encoded subunits (Fig. 2b). This finding is consistent with an essential role of Rmd9p in maintaining mitochondrial translation. While this may stem from its established role in mRNA stability, our subsequent residue-level analysis presented below further suggests that Rmd9p also contributes more directly to translation initiation.

### Rmd9p mutants are defective in mitochondrial function

**Fig. 3.**
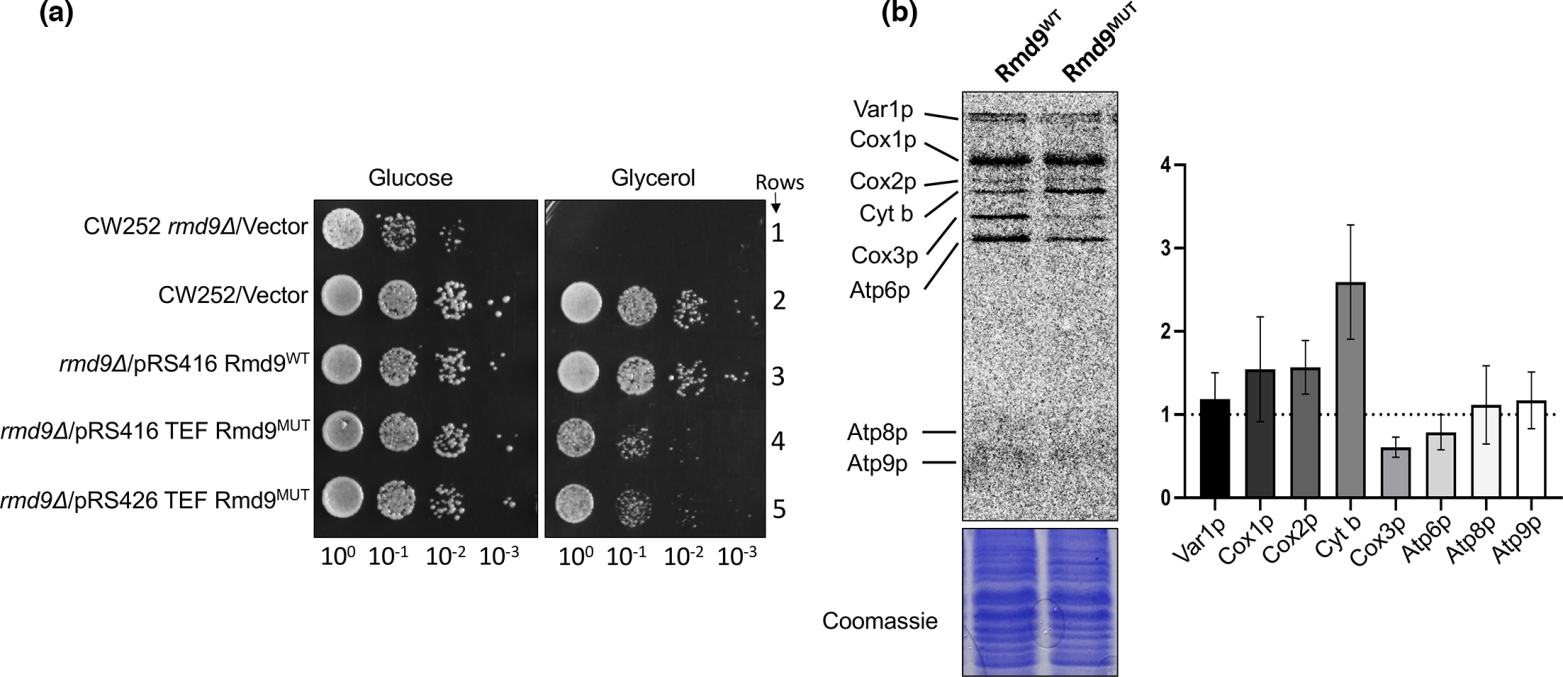
Characterization of *rmd9* mutants. (**a**) Growth analysis of yeast strains in fermentable (glucose) versus non-fermentable (glycerol) carbon source. (**b**) S^35^-Methionine labelling of mitochondrial products in wild-type and mutant rmd9 (left) and quantification of different mitochondrially encoded proteins (right); *N*=2, *n*=2.

To assess the *in vivo* function of Rmd9p, particularly of residues important for IF1-like function, we created alanine substitution mutants targeting specific amino acid residues either individually (W457, R460 and F465) or in combination (W457A/R460A or W457A/R460A/F465A). These mutants were expressed using the *rmd9* native promoter in a background where the *rmd9* gene was deleted. We observed that the single site mutants behaved similarly to the wild-type, showing no significant growth defects in a non-fermentable carbon source (Fig. S1, available in the online Supplementary Material; compare row 2 with rows 3, 4 and 6). However, the double and triple mutants (particularly the W457A/R460A/F465A triple mutant, referred to as ‘mutant’ from now on) displayed a notable growth impairment under the same conditions (Fig. S1; compare row 2 with rows 5 and 7). We noticed that the expression of the mutant was considerably lower than that of the wild-type Rmd9p (Fig. S2A). This raised a concern that the growth defect might be attributed to the reduced expression of the mutant rather than the specific mutations themselves. To address this possibility, we cloned the mutant under a strong TEF promoter, and even with the mutant expressed at much higher levels (Fig. S2B), the growth defect persisted (Fig. 3a; compare rows 3 and 4), indicating that the observed phenotype was indeed a consequence of the introduced mutations. Further, the inability of the mutant to rescue the defect even when expressed from a multicopy plasmid, pRS426 TEF (Fig. 3a, compare rows 3 and 5; Fig. S2B), validates the role of these residues in mitochondrial function.

To explore the potential cause of the growth defect observed in the mutant strain, we investigated whether the stability of mitochondrial 15S rRNA, a known function associated with Rmd9, was affected [16,24]. Using Northern blot analysis, we assessed the stability of 15S rRNA in the mutant strain. However, our results showed that there was no significant defect in the stability of 15S rRNA in the mutant strain (Fig. S3). This finding suggests that the possible IF1-like function of Rmd9p, as observed in the mutant strain, is independent of its known role in ensuring the stability of mitochondrial 15S rRNA.

### Mitochondrial translation is disturbed in the *rmd9* mutant

Mutations in IF1 are known to affect translation in bacteria [30,31]. Thus, we decided to check if mutations in *rmd9* at the residues predicted to be important for the IF1-like function influenced translation by carrying out S^35^ methionine labelling of mitochondrial translation products in wild-type and the *rmd9* deletion strain complemented with the *rmd9* mutants. As shown in Fig. 3b, the *rmd9* mutants complemented for the translation of most of the mitochondrially encoded proteins except for the COX3 and ATP6. These proteins, particularly COX3, were significantly downregulated (Fig. 3b). Although we could not ascertain precise reasons for the specific downregulation of these two proteins, this partial defect is likely sufficient to explain the slow growth of the mutant strain on non-fermentable carbon source, in line with the strong respiratory growth defects reported when COX3 or ATP6 function is severely compromised [32,33].

### Rmd9p features a substitute for IF1 function in *E. coli*

**Fig. 4.**
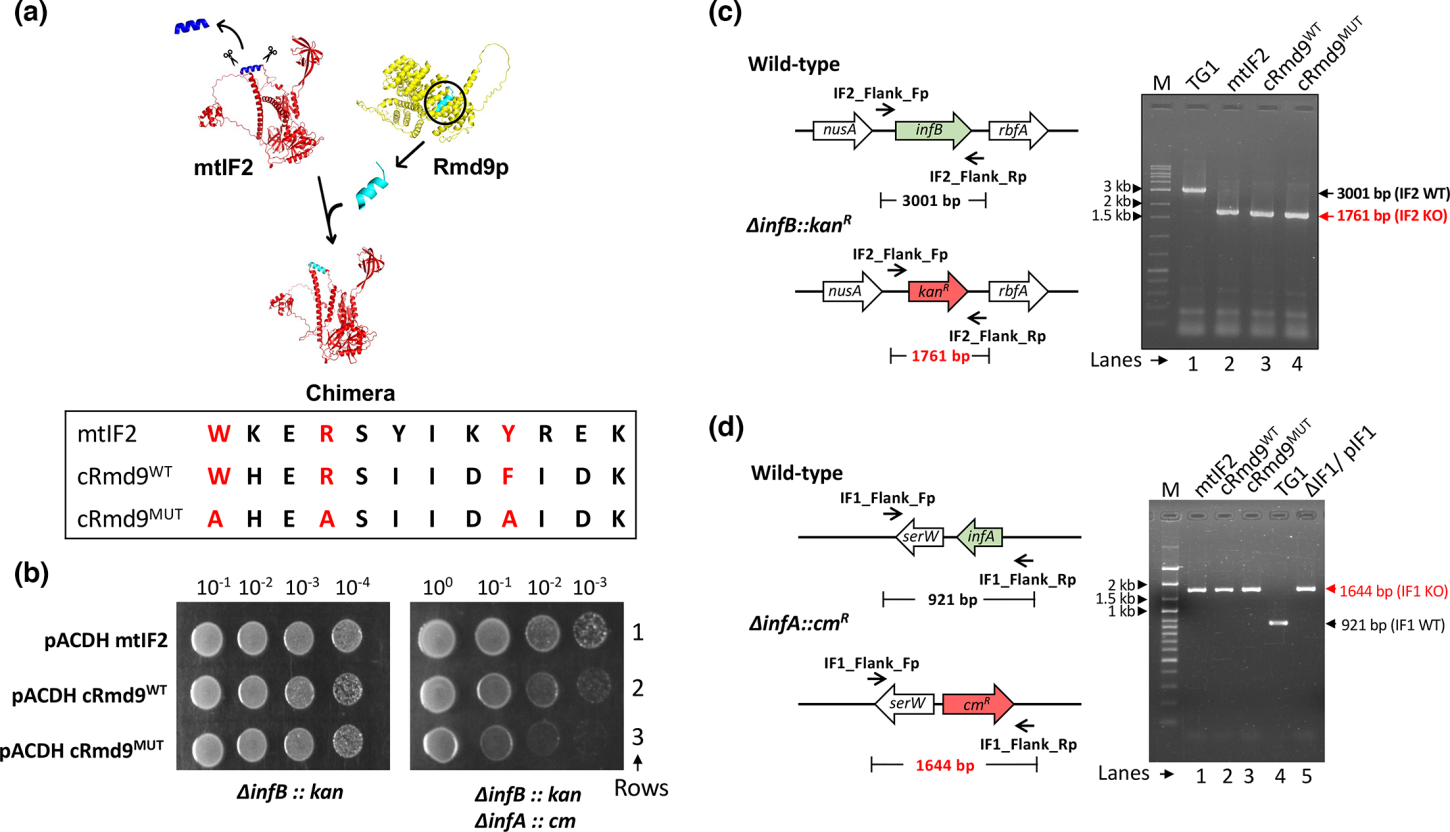
IF1-like function of Rmd9p residues in *E. coli*. (**a**) Generation of chimaeras (top panel); the chimaera was generated by replacing the bovine mtIF2 (red) sequence (in blue) with the Rmd9p (in yellow) sequence (in cyan). The sequences of mtIF2 being replaced or the Rmd9p sequence used for the chimaera (in the bottom panel). (**b**) Growth analysis using spotting assay for ΔIF2 single deletion (left) or ΔIF1 and ΔIF2 double deletion (right) under ectopic mtIF2 or chimaera support. Image taken 48 h post-spotting. (**c**) PCR verification of IF2 deletion using IF2 flanking primers. (**d**) PCR verification of IF1 deletion using IF1 flanking primers. Schematics in (**c**) and (**d**) show the amplicon sizes.

To investigate whether Rmd9p exhibits IF1-like function, we attempted IF1 deletion in *E. coli* in the presence of Rmd9p or complementing a yeast strain lacking Rmd9p with IF1. However, these efforts proved unsuccessful. Given the significant differences in the sizes of the two proteins (IF1 is a small protein of only 72 amino acids, whereas Rmd9p is a much larger protein comprising 646 amino acids), it was not unexpected that these direct approaches failed. Moreover, Rmd9 is known to play a vital role in mitochondrial RNA metabolism [16]. As an alternative approach, we generated a chimeric version of mtIF2, termed ‘cRmd9^WT^’ wherein the specific region of IF1-insert domain was replaced with a sequence of amino acids containing the identified features of Rmd9p (Fig. 4a). Subsequently, we attempted to delete both *infB* (IF2) and *infA* (IF1) genes, in the presence of chimaera as support. Remarkably, the chimeric construct was able to support the double-deletion strain in a manner mtIF2 did (Fig. 4b, rows 1 and 2, right panel). The double deletion of IF2 and IF1 was verified by PCR using *infB* locus flanking primers. A band of 3,001 bp represents the wild-type IF2 locus, and a 1,723 bp band represents the disruption of the IF2 locus by the insertion of the *kan*^R^ cassette (Fig. 4c, lanes 1 and 3). The deletion was further verified by immunoblotting using anti-IF2 antibodies (Fig. S4C). Next, IF1 deletion was verified for replacement of the *infA* locus using *cm*^R^ cassette. Using flanking primers, a band of 921 bp represents the wild-type IF1 locus and 1,644 bp band for IF1 deletion (Fig. 4d). Additionally, no amplification was observed with internal primers, ruling out the possibility of IF1 gene duplication (Fig. S5B).

We then generated a mutant form of cRmd9^WT^ termed cRmd9^MUT^, where W457, R460 and F465 residues of Rmd9p were mutated to alanine (Fig. 4a). Interestingly, this chimaera was also able to support a double deletion of *infA* and *infB*, though the complementation was not as effective as mtIF2 or cRmd9^WT^ (Fig. 4b, compare rows 1, 2 and 3, right panel). This suggests that W457, R460 and F465 residues are critical for IF1-like function of Rmd9p.

### Structural modelling of mtIF2 and chimeric proteins

**Fig. 5.**
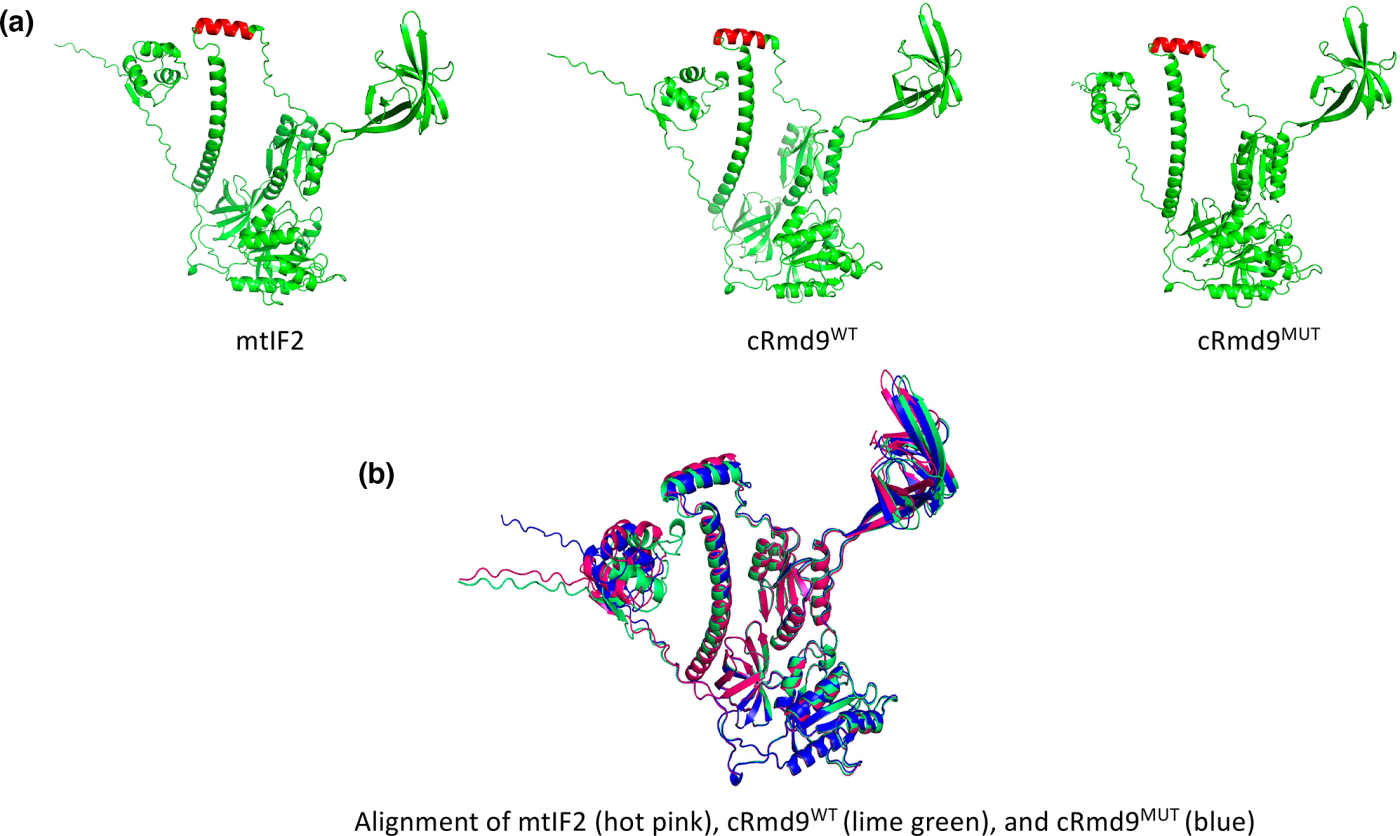
AlphaFold structure prediction of (**a**) mtIF2, cRmd9^WT^ and cRmd9^MUT^ (top panel). The insert sequence is highlighted in red. (**b**) Alignment of mtIF2 (hot pink), cRmd9^WT^ (lime green) and cRmd9^MUT^ (blue).

We performed AlphaFold predictions of mtIF2 and the chimeric proteins to look at the folding pattern of the insert domain in them. The peptide sequence from Rmd9p, in both cRmd9^WT^ and cRmd9^MUT^, was part of the insert domain helix and folds very similarly to the helix in mtIF2 (Fig. 5). This further explains why both cRmd9^WT^ and cRmd9^MUT^ can support the IF1 and IF2 double-deletion strain.

## Discussion

IF1 is one of the three essential initiation factors involved in translation initiation in bacteria. The homologues of IF1 across the three domains of life are involved in interaction with the conserved decoding nucleotides in the ribosomes (including mitoribosomes). The presence of the conserved decoding nucleotides suggests the presence of IF1-like protein(s) also in mitochondria, where translation systems have evolved differently to fulfil the role of IF1 by different proteins. In vertebrates, a 37 amino acid insert in mtIF2 and, in *T. brucei*, an extension of mtIF3 CTD participate in interactions with the decoding nucleotides [4,5, 34]. In *S. cerevisiae*, the mitochondrial translation machinery differs significantly from both the vertebrate and *T. brucei* systems, and the exact identity of an IF1-like function has remained unknown.

In this study, from among the four IF1 candidates (Rsm28p, Msc6p, Aep3p and Rmd9p), we have investigated the role of Rmd9p in serving as an IF1-like factor in *S. cerevisiae* mitochondrial translation. In yeast mitochondria, Rmd9p acts as an mRNA shuttling factor and connects mRNA maturation with translation [35]. Its significance becomes evident through its importance for mitochondrial function, and our observation further supports its essentiality in mitochondrial translation (Figs 2 and 3). The rationale behind the essential nature of Rmd9p stems from its specific functions, encompassing the stabilization and processing of mitochondrial mRNAs [16] and 3′ end processing of 15S rRNA [24,36]. And a cryo-Electron Microscopy (cryo-EM) study indicates its active involvement in the biogenesis of the mtSSU [37].

Our experiments using the Rmd9p mutant (W457A/R460A/F465A) suggest a crucial role of these residues in Rmd9 function in yeast mitochondria as the mutant does not fully complement the *rmd9* deletion strain. Intriguingly, the Rmd9p mutant does not impact overall mitochondrial translation but selectively affects translation of only a few mRNAs (Fig. 3b). While this selective impact on the translation landscape remains perplexing, our findings find resonance with mitochondrial IF3 knockout studies. The mtIF3 knockout in yeast and humans does not affect overall translation but only a few transcripts [38,39].

Rmd9p has already been shown to interact with the mtSSU through the mRNA entry proteins uS2m, uS5m, mS45 and mS46 [35]. These mitochondrial proteins lie closer to the ribosomal A-site and thus can provide Rmd9p a site to access the A-site. Hence, Rmd9p presents as an ideal candidate for IF1-like activity in *S. cerevisiae*. Nonetheless, to demonstrate its potential to serve as an IF1 candidate, we made use of a bacterial model that we used earlier [3] to demonstrate IF1-like function of the 37 amino acid insert of mammalian mtIF2 in *E. coli*. In such a system, replacement of the IF1-like insert of the mammalian mtIF2 with the corresponding sequence from Rmd9p led to the creation of a functional mtIF2 chimaera (cRmd9^WT^) that substituted for the functions of both IF1 and IF2 in *E. coli* (Fig. 4), indicating the potential of Rmd9p in contributing to the IF-1-like function in *S. cerevisiae* mitochondria. Interesting, when we mutated each of the W457, R460 and F465 residues to Ala in a triple mutant of cRmd9^WT^ (cRmd9^MUT^), it still supported *E. coli* for the dual function of IF1 and IF2, albeit with somewhat less efficient growth. This observation suggests that it is the overall structure of the 37 amino acid insert in mtIF2 that is more crucial for interaction with the decoding nucleotides than the specific amino acids in the domain. In fact, when we carried out structural modelling of the chimeric proteins (Fig. 5), it revealed that they all presented the 37 amino acid insert in a very similar configuration. To further support this observation, we decided to create chimeric proteins of mtIF2 wherein the 37 amino acid insert sequence was acquired from the Rsm28p (cRsm28) and Msc6p (cMsc6) chimaeras. Consistent with the hypothesis that it is the structural module of the ‘IF1 insert’ in mtIF2 that is more important than the sequence per se, the newly generated chimeric mtIF2 also supported the deletion of both the IF1 and IF2 protein genes from *E. coli* (Figs S4, S5 and S6). This hypothesis finds further support from a recent study in *Schizosaccharomyces pombe*, which demonstrates that its mtIF2 contains an insert domain that is structurally similar but sequence-wise distinct from the vertebrate mtIF2, yet it can compensate for the absence of IF1 [40]. Taken together, our observations suggest that multiple proteins could serve the function of IF1 in *S. cerevisiae* mitochondria. However, given the biochemical properties of Rmd9p, its localization in the mRNA binding channel close to the ribosomal A-site, we favour a major role of Rmd9p for an IF1-like function. Structural analyses would be required to further support the role of Rmd9p for an IF1-like function.

## Supplementary material

10.1099/mic.0.001689 Uncited Supplementary Material 1.

## Abbreviations

BN-PAGE: blue-native PAGE
CTE: C-terminal extension
LB: Luria–Bertani
mtSSU: mitoribosomal smaller subunit
PPR: pentatricopeptide repeat

## References

[1] Gahura O, Chauhan P, Zíková A (2022). Mechanisms and players of mitoribosomal biogenesis revealed in trypanosomatids. Trends Parasitol.

[2] Carter AP, Clemons WM, Brodersen DE, Morgan-Warren RJ, Hartsch T (2001). Crystal structure of an initiation factor bound to the 30S ribosomal subunit. Science.

[3] Gaur R, Grasso D, Datta PP, Krishna PDV, Das G (2008). A single mammalian mitochondrial translation initiation factor functionally replaces two bacterial factors. Mol Cell.

[4] Ramrath DJF, Niemann M, Leibundgut M, Bieri P, Prange C (2018). Evolutionary shift toward protein-based architecture in trypanosomal mitochondrial ribosomes. Science.

[5] Kummer E, Leibundgut M, Rackham O, Lee RG, Boehringer D (2018). Unique features of mammalian mitochondrial translation initiation revealed by cryo-EM. Nature.

[6] Chicherin IV, Baleva MV, Levitskii SA, Dashinimaev EB, Krasheninnikov IA (2020). Initiation factor 3 is dispensable for mitochondrial translation in cultured human cells. Sci Rep.

[7] Tibbetts AS, Oesterlin L, Chan SY, Kramer G, Hardesty B (2003). Mammalian mitochondrial initiation factor 2 supports yeast mitochondrial translation without formylated initiator tRNA. J Biol Chem.

[8] Vambutas A, Ackerman SH, Tzagoloff A (1991). Mitochondrial translational-initiation and elongation factors in *Saccharomyces cerevisiae*. Eur J Biochem.

[9] Atkinson GC, Kuzmenko A, Kamenski P, Vysokikh MY, Lakunina V (2012). Evolutionary and genetic analyses of mitochondrial translation initiation factors identify the missing mitochondrial IF3 in S. cerevisiae. Nucleic Acids Res.

[10] Franco LVR, Moda BS, Soares MAKM, Barros MH (2019). Msc6p is required for mitochondrial translation initiation in the absence of formylated Met-tRNA^fMet^. FEBS J.

[11] Aibara S, Singh V, Modelska A, Amunts A (2020). Structural basis of mitochondrial translation. Elife.

[12] Herbert CJ, Golik P, Bonnefoy N (2013). Yeast PPR proteins, watchdogs of mitochondrial gene expression. RNA Biol.

[13] Luo Y, Wang Y, Huang Y (2021). *Schizosaccharomyces pombe* Ppr10 and Mpa1 together mediate mitochondrial translational initiation. J Biol Chem.

[14] Bridgers JB, Carlström A, Sherpa D, Couvillion MT, Rovšnik U (2025). Translational activators align mRNAs at the small mitoribosomal subunit for translation initiation. Nat Struct Molecul Biol.

[15] Lee C, Tibbetts AS, Kramer G, Appling DR (2009). Yeast AEP3p is an accessory factor in initiation of mitochondrial translation. J Biol Chem.

[16] Nouet C, Bourens M, Hlavacek O, Marsy S, Lemaire C (2007). Rmd9p controls the processing/stability of mitochondrial mRNAs and its overexpression compensates for a partial deficiency of oxa1p in *Saccharomyces cerevisiae*. Genetics.

[17] Williams EH, Butler CA, Bonnefoy N, Fox TD (2007). Translation initiation in *Saccharomyces cerevisiae* mitochondria: functional interactions among mitochondrial ribosomal protein Rsm28p, initiation factor 2, methionyl-tRNA-formyltransferase and novel protein Rmd9p. Genetics.

[18] Williams EH, Bsat N, Bonnefoy N, Butler CA, Fox TD (2005). Alteration of a novel dispensable mitochondrial ribosomal small-subunit protein, Rsm28p, allows translation of defective COX2 mRNAs. Eukaryot Cell.

[19] Lipinski KA, Puchta O, Surendranath V, Kudla M, Golik P (2011). Revisiting the yeast PPR proteins--application of an Iterative Hidden Markov Model algorithm reveals new members of the rapidly evolving family. Mol Biol Evol.

[20] Moda BS, Ferreira-Júnior JR, Barros MH (2016). Partial suppression of the respiratory defect of qrs1/her2 glutamyl-tRNA amidotransferase mutants by overexpression of the mitochondrial pentatricopeptide Msc6p. Curr Genet.

[21] Ellis TP, Helfenbein KG, Tzagoloff A, Dieckmann CL (2004). Aep3p stabilizes the mitochondrial bicistronic mRNA encoding subunits 6 and 8 of the H+-translocating ATP synthase of *Saccharomyces cerevisiae*. J Biol Chem.

[22] Barros MH, Tzagoloff A (2017). Aep3p-dependent translation of yeast mitochondrial *ATP8*. Mol Biol Cell.

[23] Hillen HS, Markov DA, Wojtas ID, Hofmann KB, Lidschreiber M (2021). The pentatricopeptide repeat protein Rmd9 recognizes the dodecameric element in the 3’-UTRs of yeast mitochondrial mRNAs. Proc Natl Acad Sci.

[24] Singh J, Singh S, Emam EAF, Varshney U (2024). Role of Rmd9p in 3’-end processing of mitochondrial 15S rRNA in Saccharomyces cerevisiae. Mitochondrion.

[25] Hemsley A, Arnheim N, Toney MD, Cortopassi G, Galas DJ (1989). A simple method for site-directed mutagenesis using the polymerase chain reaction. Nucleic Acids Res.

[26] Mumberg D, Müller R, Funk M (1995). Yeast vectors for the controlled expression of heterologous proteins in different genetic backgrounds. Gene.

[27] Ito H, Fukuda Y, Murata K, Kimura A (1983). Transformation of intact yeast cells treated with alkali cations. J Bacteriol.

[28] Gouget K, Verde F, Barrientos A (2008). In vivo labeling and analysis of mitochondrial translation products in budding and in fission yeasts. *Methods Mol Biol*.

[29] Timón-Gómez A, Pérez-Pérez R, Nyvltova E, Ugalde C, Fontanesi F (2020). Protocol for the analysis of yeast and human mitochondrial respiratory chain complexes and supercomplexes by blue native electrophoresis. STAR Protoc.

[30] Croitoru V, Bucheli-Witschel M, Hägg P, Abdulkarim F, Isaksson LA (2004). Generation and characterization of functional mutants in the translation initiation factor IF1 of Escherichia coli. Eur J Biochem.

[31] Croitoru VV, Bucheli-Witschel M, Isaksson LA (2005). In vivo involvement of mutated initiation factor IF1 in gene expression control at the translational level. FEBS Lett.

[32] Rak M, Tetaud E, Godard F, Sagot I, Salin B (2007). Yeast cells lacking the mitochondrial gene encoding the ATP synthase subunit 6 exhibit a selective loss of complex IV and unusual mitochondrial morphology. J Biol Chem.

[33] Weiss-Brummer B, Guba R, Haid A, Schweyen RJ (1979). Fine structure of OXI1, the mitochondrial gene coding for subunit II of yeast cytochrome c oxidase. Curr Genet.

[34] Yassin AS, Haque ME, Datta PP, Elmore K, Banavali NK (2011). Insertion domain within mammalian mitochondrial translation initiation factor 2 serves the role of eubacterial initiation factor 1. Proc Natl Acad Sci U S A.

[35] Singh AP, Salvatori R, Aftab W, Kohler A, Carlström A (2020). Molecular connectivity of mitochondrial gene expression and OXPHOS biogenesis. Mol Cell.

[36] Anikin M, Henry MF, Hodorova V, Houbaviy HB, Nosek J (2025). Mitochondrial mRNA and the small subunit rRNA in budding yeasts undergo 3′-end processing at conserved species-specific elements. RNA.

[37] Harper NJ, Burnside C, Klinge S (2023). Principles of mitoribosomal small subunit assembly in eukaryotes. Nature.

[38] Kuzmenko A, Derbikova K, Salvatori R, Tankov S, Atkinson GC (2016). Aim-less translation: loss of Saccharomyces cerevisiae mitochondrial translation initiation factor mIF3/Aim23 leads to unbalanced protein synthesis. Sci Rep.

[39] Remes C, Khawaja A, Pearce SF, Dinan AM, Gopalakrishna S (2023). Translation initiation of leaderless and polycistronic transcripts in mammalian mitochondria. Nucleic Acids Res.

[40] Luo Y, Bähler J, Huang Y (2025). The insertion domain of Mti2 facilitates the association of mitochondrial initiation factors with mitoribosomes in *Schizosaccharomyces pombe*. Biomolecules.

[41] Saint-Georges Y, Bonnefoy N, di Rago JP, Chiron S, Dujardin G (2002). A pathogenic cytochrome b mutation reveals new interactions between subunits of the mitochondrial bc1 complex. J Biol Chem.

[42] Sambrook J, Fritsch ER, Maniatis T (1989). Molecular Cloning: A Laboratory Manual 2nd ed.

