# Supplementary material for: Transplantation of Saccharomyces cerevisiae Rmd9p peptide into mammalian mitochondrial IF2 substitutes for the IF1 function in Escherichia coli
Source: Microbiology (Reading). 2026 Mar 27;172(3):001689. doi: 10.1099/mic.0.001689 (PMC13030852; doi:10.1099/mic.0.001689)
Supplement: Uncited Supplementary Material 1. [file mic-172-01689-s001.pdf]

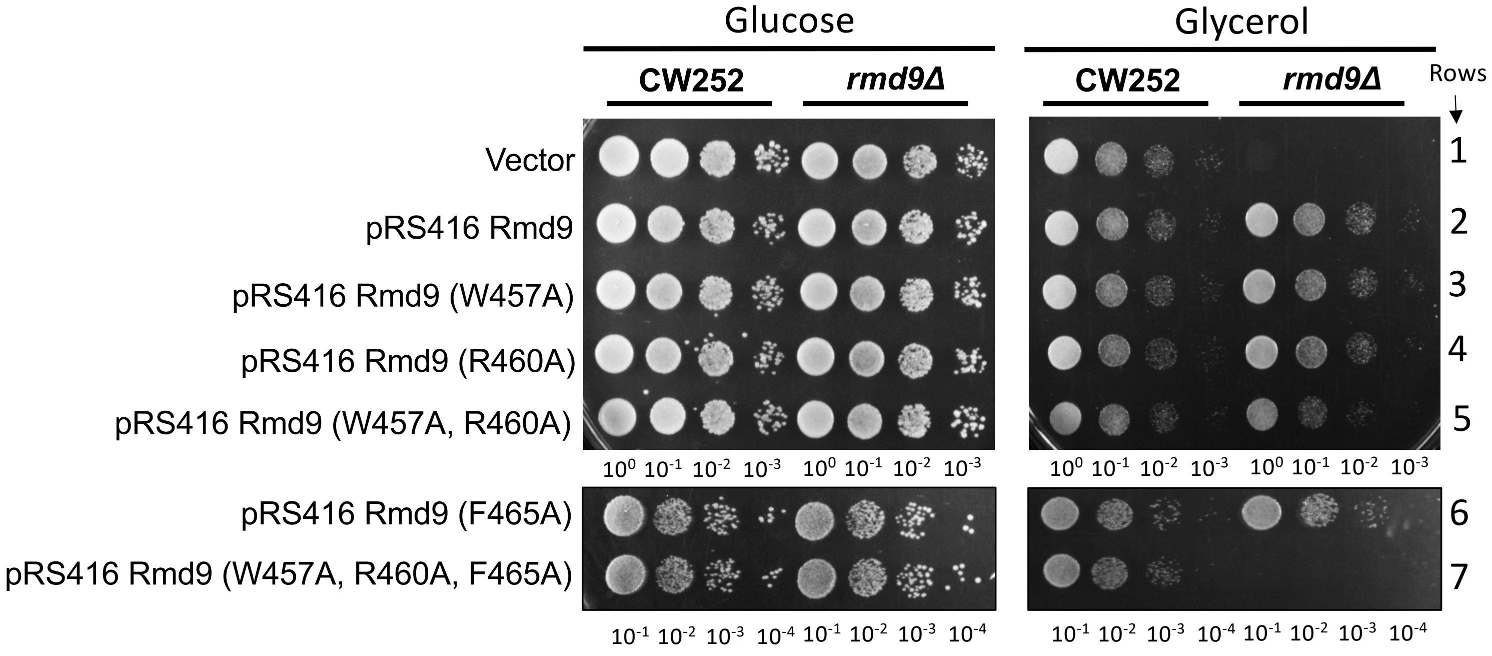

Figure S1.

Growth analysis using spotting assays for different *rmd9* mutants. The mutants were expressed under *rmd9* native promoter.

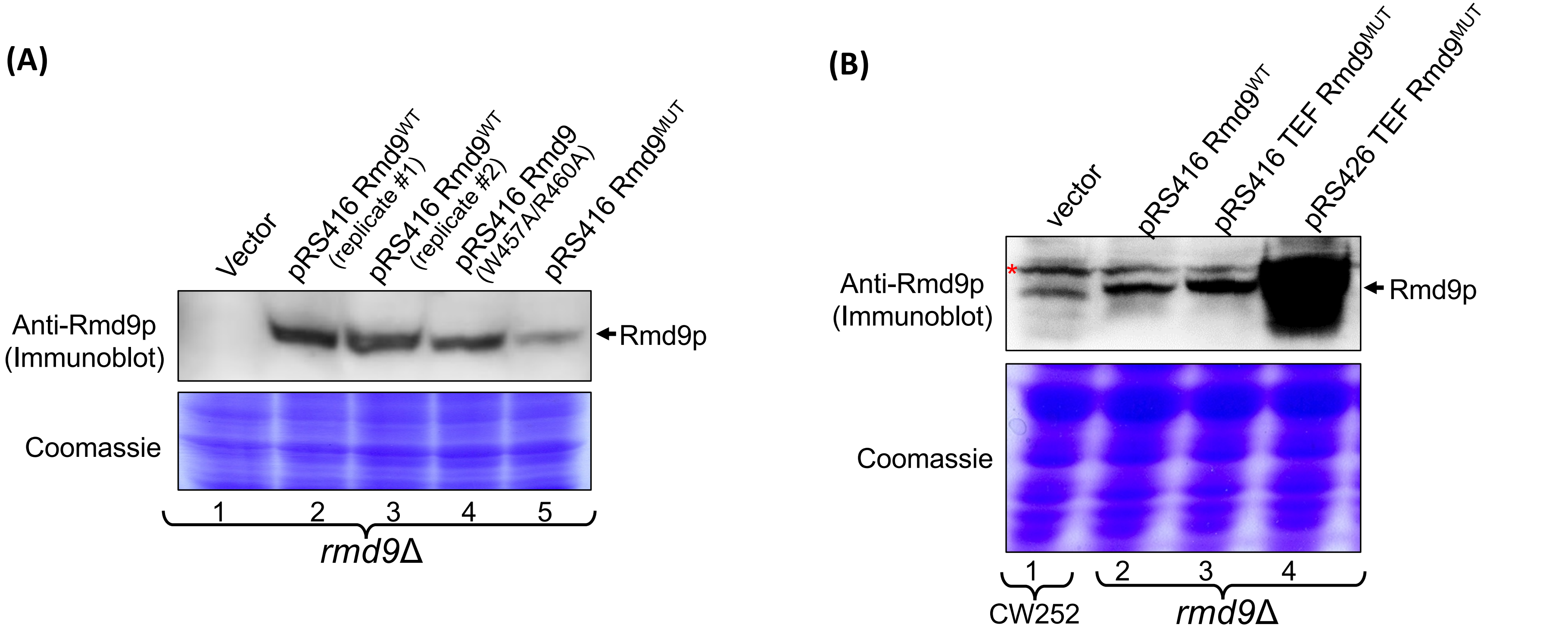

Figure S2.

(A and **B)** Immunoblot analysis of the Rmd9p [WT; W457A/R460A; and MUT (W457A/R460A/F465A)] expressed in *rmd9*Δ (*panel* **A**) or CW252 (*panel* **B**, *lane 1*) and *rmd9*Δ (*panel* **B**, *lanes 2-4*) strains under TEF promoter from single copy (pRS416 TEF) or multicopy (pRS426 TEF) plasmids. Strain backgrounds are as indicated below the Coomassie stained gets in *panels* **A** and **B**. Red asterisk in *panel* **B** indicates a nonspecific band.

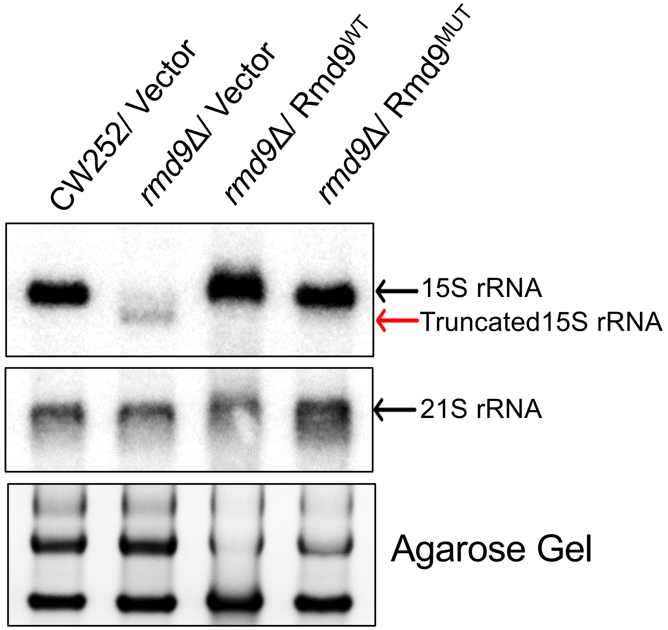

Figure S3.

Northern blot analysis of total RNA from CW252, CW252Δ*rmd9* and CW252Δ*rmd9* complemented with wild type or the mutant proteins, using 15S rRNA probe. 21S rRNA and RNA agarose gel were used as a loading controls.

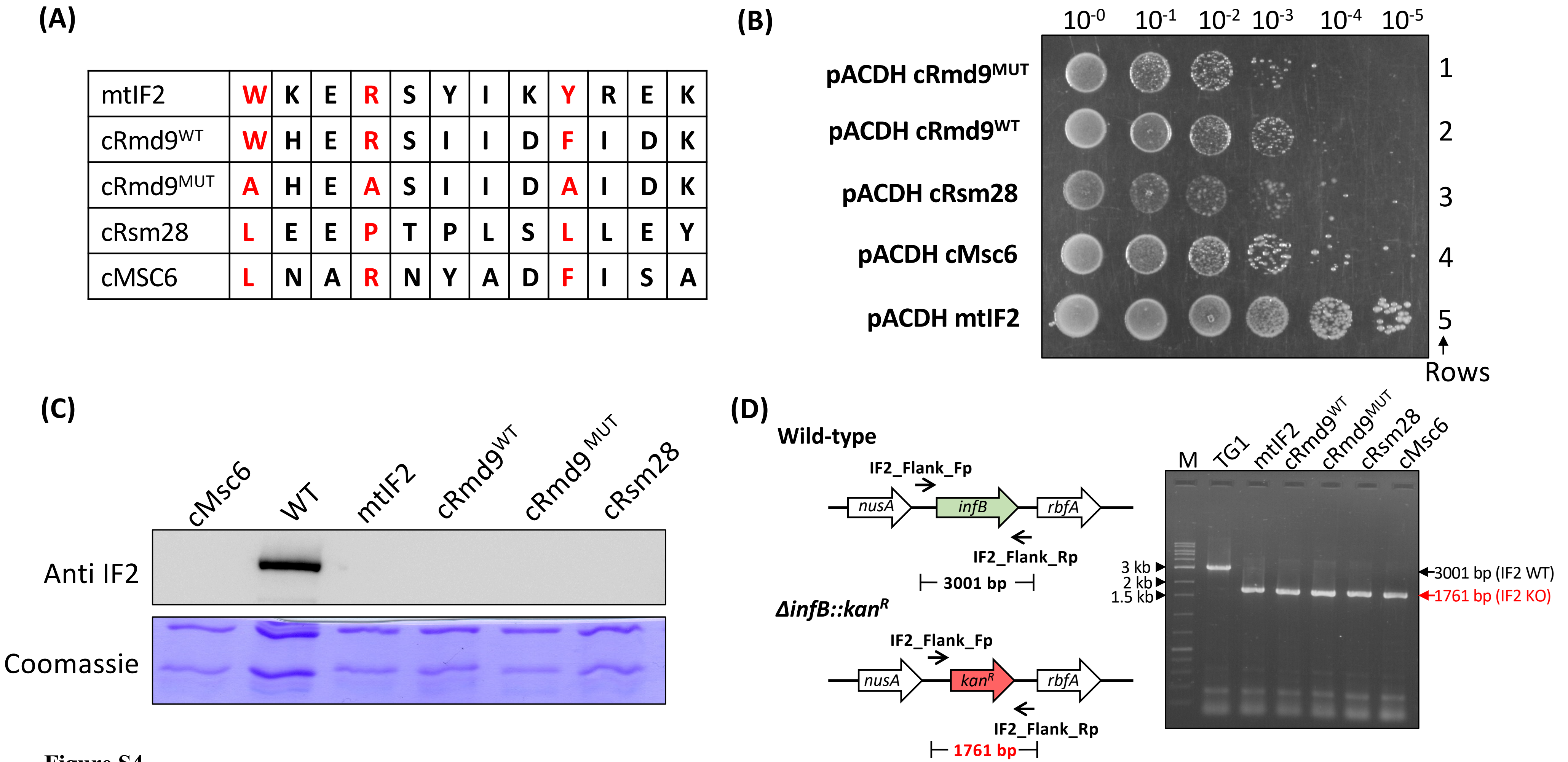

mtIF2	W	K	E	R	S	Y	I	K	Y	R	E	K
cRmd9^{WT}	W	H	E	R	S	I	I	D	F	I	D	K
cRmd9^{MUT}	A	H	E	A	S	I	I	D	A	I	D	K
cRsm28	L	E	E	P	T	P	L	S	L	L	E	Y
cMSC6	L	N	A	R	N	Y	A	D	F	I	S	A

Figure S4.

(A) Insert sequences used for generating different chimeras to replace mtIF2 chimera. **(B)** Growth analysis using spotting assay for ΔIF1, ΔIF2 double deletion under ectopic mtIF2 or the chimera support. Image taken 72h post spotting. **(C)** Confirmation of IF2 deletion using western blot for endogenous IF2 under the support of different chimeras (top panel) and the Coomassie stained gel portion (bottom). **(D)** PCR verification of IF2 deletion using IF2 flanking primers for various chimeras. The panel in the left shows the schematic for the expected amplicon sizes. The panel on the right is the agarose gel. Relevant DNA size markers are indicated (M).

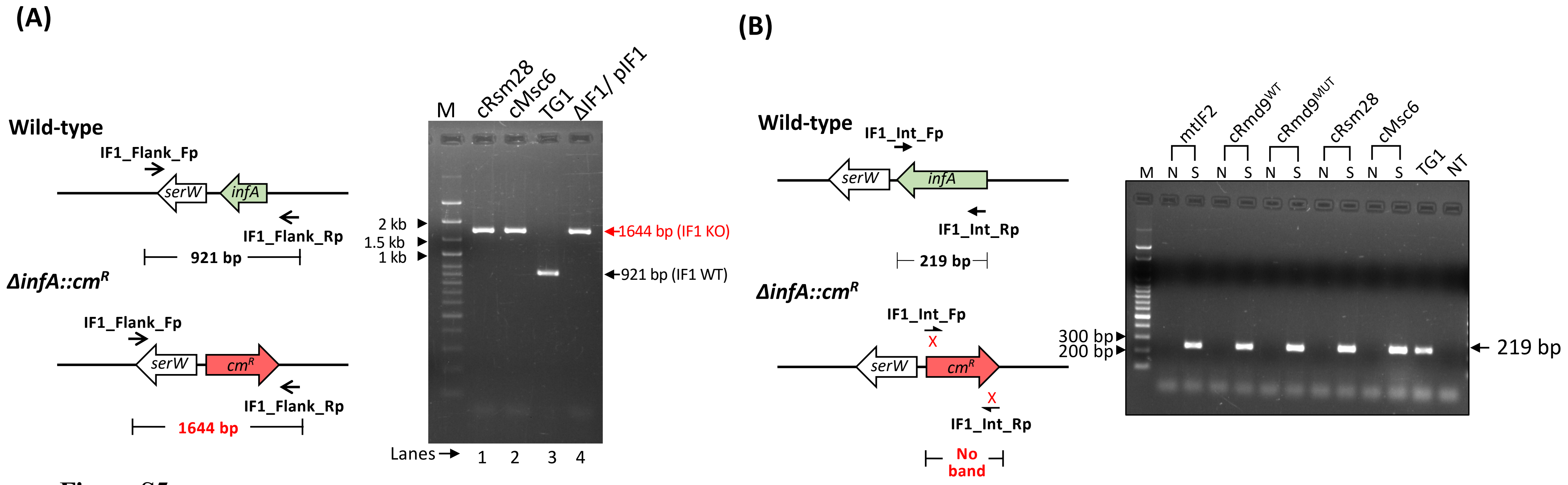

Figure S5.

(A) PCR verification of IF1 deletion in ΔIF2 strains supported by Rsm28p and Msc6p derived chimeras using IF1 flanking primers. Schematic for the expected amplicon sizes is shown in the left panel, and the agarose gel is shown in the right panel. Relevant DNA size markers are as indicated (M). **(B)** PCR verification of IF1 deletion in ΔIF2 strains supported by various chimeras using IF1 internal primers. Schematic for the expected amplicon sizes is shown in the left panel, and the agarose gel is shown in the right panel. Relevant DNA size markers are as indicated (M). Lanes labelled as 'N' only has templates derived from double deletion strains, while lanes labelled as 'S' were spiked with template from a wild type strain.

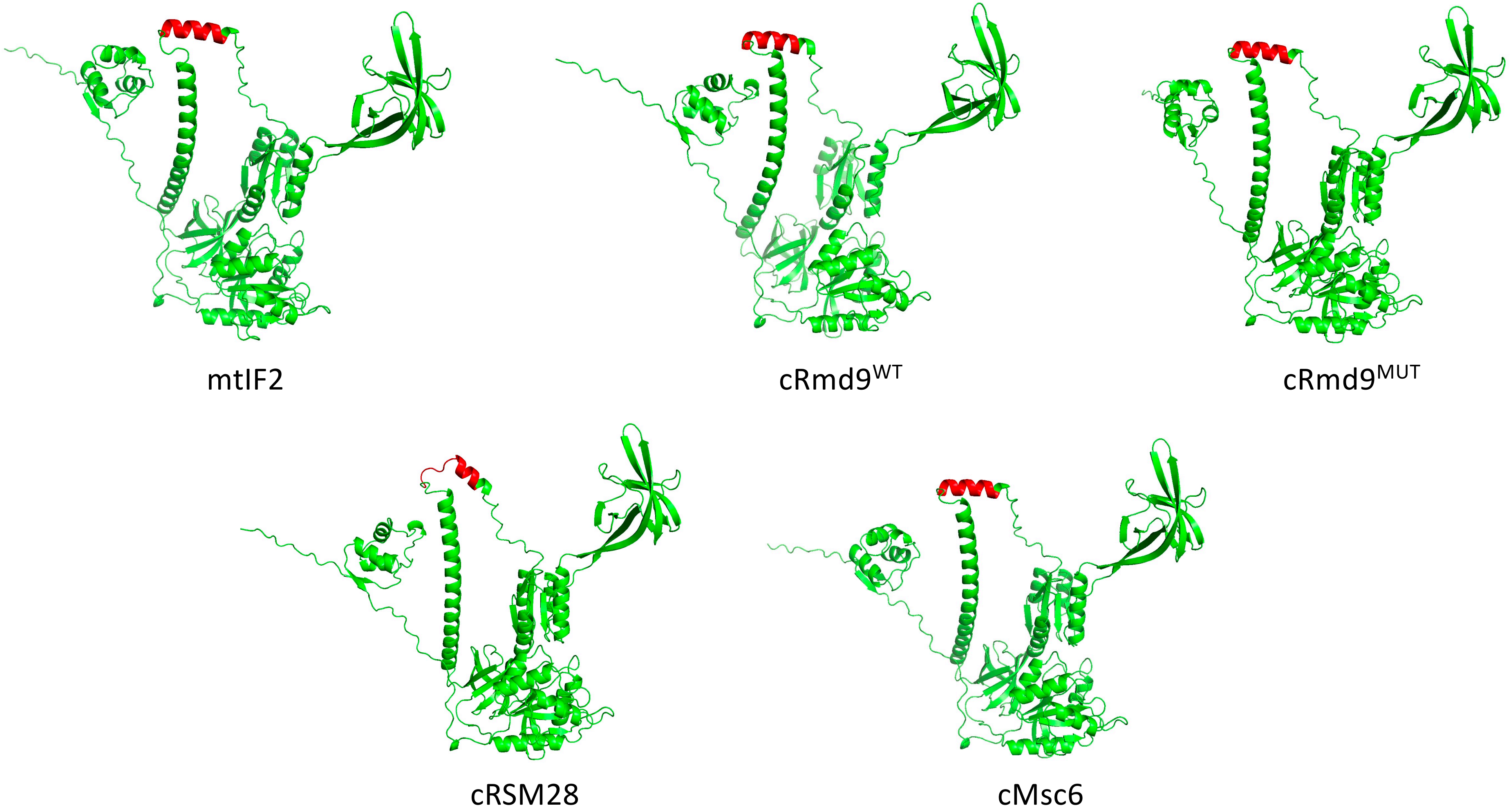

Figure S6.

AlphaFold structure predictions of mtIF2, cRmd9^{WT}, cRmd9^{MUT} cRsm28, and cMsc6 with the insert sequence is highlighted in red.
